# Technological and Theoretical Aspects for Testing Electroporation on Liposomes

**DOI:** 10.1155/2017/5092704

**Published:** 2017-03-14

**Authors:** Agnese Denzi, Elena della Valle, Gianluca Esposito, Lluis M. Mir, Francesca Apollonio, Micaela Liberti

**Affiliations:** ^1^Center for Life Nano Science@Sapienza, Istituto Italiano di Tecnologia, 00161 Rome, Italy; ^2^Department of Information Engineering, Electronics and Telecommunication (DIET), University of Rome “La Sapienza”, 00184 Rome, Italy; ^3^Vectorology and Antitumor Therapies, UMR 8203, CNRS, Univ. Paris-Sud, Gustave Roussy, Université Paris-Saclay, 94805 Villejuif, France

## Abstract

Recently, the use of nanometer liposomes as nanocarriers in drug delivery systems mediated by nanoelectroporation has been proposed. This technique takes advantage of the possibility of simultaneously electroporating liposomes and cell membrane with 10-nanosecond pulsed electric fields* (nsPEF)* facilitating the release of the drug from the liposomes and at the same time its uptake by the cells. In this paper the design and characterization of a 10* nsPEF* exposure system is presented, for liposomes electroporation purposes. The design and the characterization of the applicator have been carried out choosing an electroporation cuvette with 1 mm gap between the electrodes. The structure efficiency has been evaluated at different experimental conditions by changing the solution conductivity from 0.25 to 1.6 S/m. With the aim to analyze the influence of device performances on the liposomes electroporation, microdosimetric simulations have been performed considering liposomes of 200 and 400 nm of dimension with different inner and outer conductivity (from 0.05 to 1.6 S/m) in order to identify the voltage needed for their poration.

## 1. Introduction

In the last years, the application of electric pulses to induce biological effects proved to be an effective tool in different fields (e.g., cancer treatment, gene transfer, and electrofusion) [1–4]. The main biological effect is related to an increment in membranes permeability with a rearrangement of phospholipid bilayer and a consequent formation of pores that can permit the uptake of molecules, genes, and drugs through the cell membrane [1, 5, 6]. This structural membrane rearrangement seems to be connected with an increase of the transmembrane potential with respect to a resting condition [7, 8].

Recently, the authors proposed a technique combining the application of nanosecond pulsed electric fields* (nsPEFs)* with liposomes as nanocarriers in drug delivery applications [9]. The possibility of simultaneously porating cell membrane and liposomes with nanometer dimension has been numerically demonstrated in the case of the delivery of a single electric pulse of 10 ns duration and few MV/m of amplitude. This could open the way to a liposomal drug delivery system mediated by nanoelectroporation, facilitating drugs release from the liposomes to the cell.

The application to cells of one or several* nsPEF* of 10 ns duration is very common in nanoporation [10–15]. Different types of applicators and devices can be used without compromising the shape of the applied pulse [16–21]. Moreover, with the advances in micro- and nanofabrication techniques, some microdevices are proposed for nanosecond exposure, integrated also with a microfluidic system [16, 19, 22, 23]. A typical applicator is a standard electroporation cuvette consisting of two plate parallel electrodes with a gap of 4, 2, or 1 mm [12, 13, 18, 20]. In particular, the 1 mm gap cuvette permits generating higher values of the electric field in the solution, but in such cases the matching with the 50 Ω of the pulse generator can be critical [17, 20].

However, the choice of a 10 ns pulse, due to its frequency content (first lobe at around 80–100 MHz depending on the rise and fall times), allows comfortably selecting the cuvette as applicator, even if the matching conditions of the structure need to be evaluated. Conversely, for shorter pulse durations (i.e., 3 ns and 1 ns), the complication of a broadband matching, up to GHz region, becomes necessary otherwise the cuvette will cause a significant distortion of the trapezoidal signal.

The use of a standard cuvette is also advantageous in the case of nanometer liposomes exposure related to the necessity to detect a fluorescent release. Indeed, due to their small dimensions, liposomes visibility under a microscope cannot be possible. In this case the fluorescent measurement is done with a spectrofluorimeter reader able to detect fluorescent molecule concentrations. This technique requires a minimum volume of solution, easily collected from the electroporation cuvette [24, 25].

In this paper, we propose a methodology for the exposure of liposomes with dimension in the nanometer scale (in particular 200 and 400 nm liposomes) in terms of design of the exposure system combined with theoretical evaluation of the electric field threshold needed to porate the liposomes. As a first step, we propose an integrated approach to deal with experimental and modeling aspects, with the fundamental role of understanding and interpreting biological results. Once the specifications needed by our application were clarified, with particular care to the connection with the generator, the cuvette holder and the transition to the coaxial connector have been designed. A complete frequency characterization of the final structure, both with numerical modeling and measurements, has been done in order to understand the cuvette response under different experimental conditions. After the characterization of the exposure system, a microdosimetry model of the liposomes was also developed to approach the electric field needed for the poration considering the limitations imposed by the real experimental setup.

## 2. Materials and Methods

### 2.1. Integrated Approach for Biological Understanding

**Figure 1:**
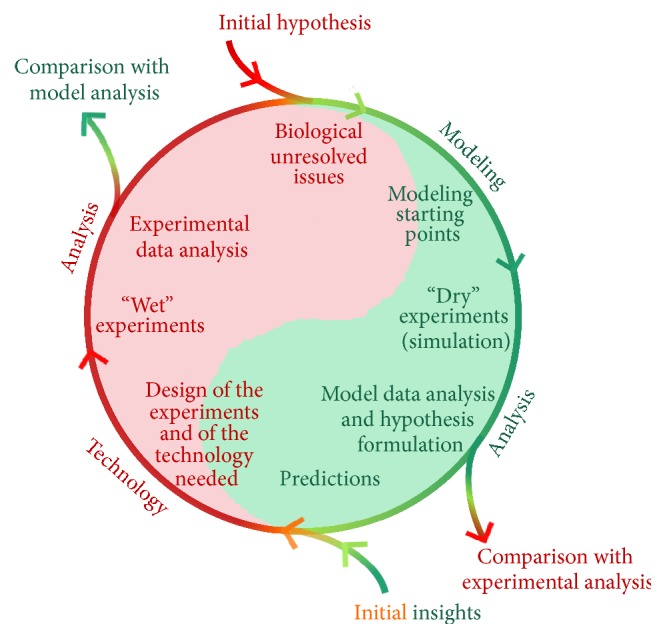
Schematic of the integrated approach necessary to understand the biological results: the cycle points out the importance of a continuous exchange between the experimental and modeling aspects.

The researchers agree that, in biological issues resolution, the cooperation between experiments and modeling must be considered as a fundamental step [12, 16, 27, 28]. In particular, this aspect is critical and significant when one wants to investigate the interactions between the electromagnetic fields and the biological objects. In Figure 1 a readapted version of the Kitano cycle proposed for system biology [29, 30] has been reported and applied to our issue.

The basic concept is the possibility of beginning the cycle at different points of the experimental and model parts. Wherever there is an output from the cycle, an improvement in our biological knowledge is attained. Two different strategies exist:* “Model Driven”* and* “Experiment Driven.”* In the first one, corresponding to the green part of the cycle, the model part drives the experiments generating a prediction. In the latter, associated with the red part of the cycle, the process begins from the experimentation with useful information to build a model able to explain what observed. When the access to the cycle begins from a particular biological hypothesis or issue (red arrow in the Figure 1 with reported initial hypothesis) or comes from the previous step of experimental data analysis, starting points and data for the model are provided. It is possible to build a simulation (that is called the “dry” experiment) to take into account the biological aspects, and this can be done at different levels of complexity (atomistic level and from the cell (microlevel) to the tissue and organs levels (macrolevel)) [5, 27, 31–35]. The simulation results can be used to formulate hypothesis about the biological issues or to simulate the behavior of a particular device that then can be used for the experiments. At this point it is possible to get out from the cycle and compare the modeling results with the experimental data analysis or alternatively keep cycling to use the prediction to set up a “wet” experiment. To set up a “wet” experiment, a technological step is needed starting from modeling results. Next, the analysis of the “wet” experimental results is provided, and a direct comparison is possible with modeling outcomes getting out from the cycle. Alternatively, this analysis can be reused as a starting point for a new or upgraded model in a continuous exchange of information and knowledge.

### 2.2. Towards “Wet” Experiments for Electroporation on Liposomes

#### 2.2.1. Starting Points

The starting points for electroporation applications regard the feature of the pulse and the target of the stimulation.

**Figure 2:**
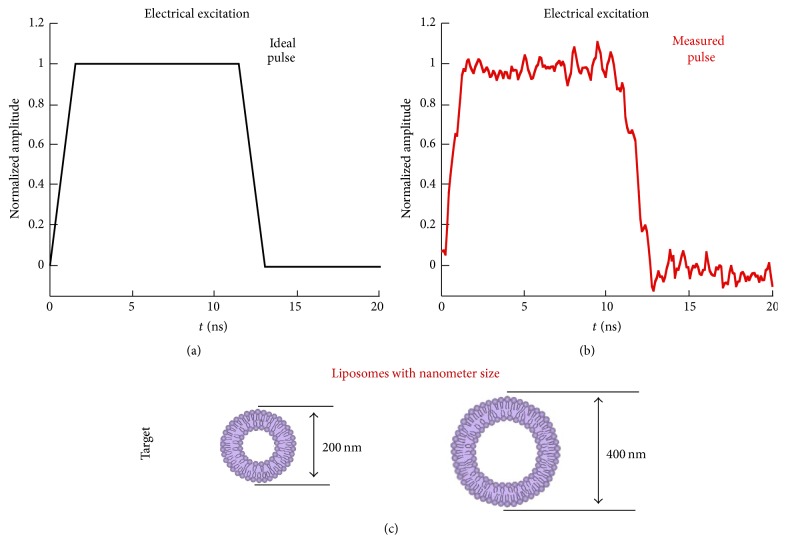
Electrical stimulus (ideal and measured pulses) and target for nanoelectroporation of liposomes.

The study of liposomes poration can be the first step for the use of this kind of nanocarriers in drug delivery applications driven by nanoelectroporation. In [9], the authors numerically demonstrated the possibility of porating liposomes of dimension up to 400 nm with a 10 ns pulse. Starting from these results, here an ideal 10 ns pulse (Figure 2(a)) has been considered in the simulations as a good model of the one that our high voltage 50 Ω pulse generator (High Voltage Pulse Generator FPG 10-1NM10) is able to produce (i.e., Figure 2(b), example of a measured pulse). The target is represented by liposomes of 200 and 400 nm of diameter (see Figure 2(c)).

The selected stimulus and target (coming from the prediction in [9]) drove the choice for the design of the experiments and of the experimental device (following the cycle in Figure 1).

In case of the 10 ns pulse, the spectral content is mainly contained in the first lobe (from 80 to 100 MHz) [9] and hence a standard cuvette for electroporation can be selected as applicator without causing significant distortion on the pulse shape [13, 18, 20]. However, the necessity to reach high electric field values for the poration of such small liposomes has led to the choice of a cuvette with 1 mm gap distance between the electrodes (1002561E, BioGenerica, ITA) and a particular attention has been taken regarding the connection of this cuvette to our 50 Ω pulse generator.

#### 2.2.2. Design of the Applicator

**Figure 3:**
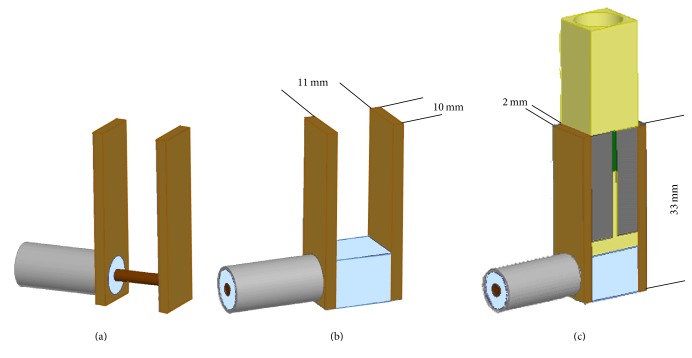
Modeling of the cuvette connection to the 50 Ω generator.

In this paragraph, the design of the structure for the connection with the 50 Ω generator has been reported. In particular, the final proposed structure is shown in Figure 3. The idea is not to directly connect the coaxial cable to the cuvette [20] but to use the coaxial cable as a lateral feed of the cuvette holder.

The device consists of two brass electrodes with area of 33 × 10 mm^2^ and a thickness of 2 mm (Figure 3). The gap between the electrodes is 11 mm and permits the perfect insertion of the electroporation cuvette between them. The central pin of the coaxial cable is connected with one of the two parallel electrodes (Figure 3(a)) and the other is connected to the external sock (Figure 3(b)). To mechanically stabilize the structure but also to avoid a central pin without any protection, a box of Teflon is placed at the bottom of the structure (Figure 3(b)). In Figure 3(c) the complete structure with the cuvette placed between the electrodes has been reported. The cuvette is a standard one (1002561E, BioGenerica, ITA), with dimension of active electrodes of 10 × 8 mm^2^ (*A*) and gap between them of 1 mm. In the first simulations, the cuvette is filled with a solution with a conductivity of 0.25 S/m calculated in order to obtain an impedance of 50 Ω, following the formulation in [20] for lower frequencies where the resistive behavior is predominant, *Z*_cuvette_ = *d*/(*Aσ*_ext_). Furthermore, the characterization of the applicator response, for different conductivity values of the solution filling the cuvette, has been carried out in order to understand the effect of this parameter on the structure performances. In particular, also other two different values have been considered, 0.55 S/m and 1.6 S/m. It is worth noticing that the impedance of the cuvette depends on external conductivity values (*σ*_ext_) and hence also the matching with the 50 Ω generator. The change of conductivity determines, at lower frequencies, a variation of the impedance from 50 Ω (for 0.25 S/m,) to around 23 Ω and 8 Ω for 0.55 S/m and 1.6 S/m, respectively. As a first step the cuvette is considered fully filled to guarantee the maximum exposure volume and to avoid sparks in air with higher input voltage.

To evaluate the performance both in frequency and time domains, the complete structure has been numerically simulated with finite element software ANSYS HFSS 2015. A wave port, applied to feed the coaxial cable, is used as input in the frequency domain; conversely a lumped port with the pulse as excitation is applied in the time domain.

#### 2.2.3. Realization and Characterization of the Applicator

After the design and characterization of the structure by numerical simulations, the fabrication process has been performed following the specifications obtained from the model results.

**Figure 4:**
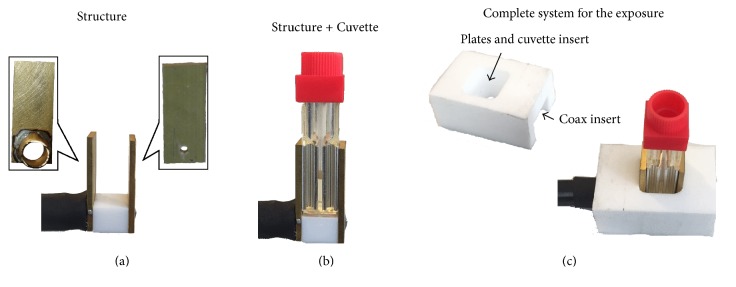
Realized structure.

The electrodes are made in brass; the one connected with the pin of the coaxial cable has been perforated to allow the insertion of the pin and an easy weld. The second one has a hole of larger dimension for the insertion of the dielectric. The connection with the external sock is made wrapping a cylinder of brass previously welded to the second electrode in proximity of the hole (Figure 4(a)). The central pin has been passed through a box of Teflon to be covered. This step has been made also to obtain a stronger mechanical stability during the application of the pulse. In Figure 4(b) the complete structure with the cuvette is reported. The cuvette can be perfectly inserted between the two electrodes and can lean on the Teflon box. In order to guarantee even more mechanical stability and a good contact between the holder and the cuvette electrodes, a further Teflon cover has been designed (Figure 4(c)). The cover presents hollows to insert the cuvette and the coaxial cable.

Because the cuvette is disposable, the use of a structure as the one here proposed can be very useful instead of a direct connection of the coaxial cable with the electrodes of the cuvette to be renewed at any cuvette usage [20].

In order to characterize the structure, measurements in the frequency domain have been carried out with an Agilent Technologies PNA Network Analyzer E8363C (10 MHz–40 GHz) with the cuvette filled with solutions at different conductivity values (0.25, 0.55, and 1.6 S/m); hence it is possible to obtain the efficiency of the structure under different experimental conditions. In this first analysis, the solutions are just made of sodium chloride 0.9% with a different dilution of DI water to obtain the desired electrical conductivity of the solution. The conductivity values of the solutions were confirmed with measurement with a Precision LCR Meter E4980A from Agilent.

#### 2.2.4. Microdosimetry Model of Nanoelectroporation of Liposomes

Due to the different experimental conditions, selectable when using liposomes (e.g., dimensions, inner conductivity, and external conductivity), a microdosimetry model has been proposed in order to understand the voltage needed at the generator for liposomes nanoporation. 2D numerical simulations have been carried out using the software Comsol Multiphysics v. 5.0. The 2D models consist of a rectangular box with dimensions of 70 *μ*m × 100 *μ*m representing the extracellular medium, in which a liposome with diameter of 200 nm or 400 nm and membrane thickness of 5 nm has been placed. The electric field has been applied with two electrodes on the box boundaries; in particular on the upper and the lower edges the excitation of 10 ns pulse and the ground are, respectively, modeled. The other boundaries are electrically insulated. The pulsed electric field amplitude has a duration of 10 ns (rise and fall time of 1.5 ns and *t*_on_ = 10 ns).

All the electrical and geometrical parameters of the model are reported in Table 1.

## 3. Results and Discussion

### 3.1. Applicator Frequency Performances

**Figure 5:**
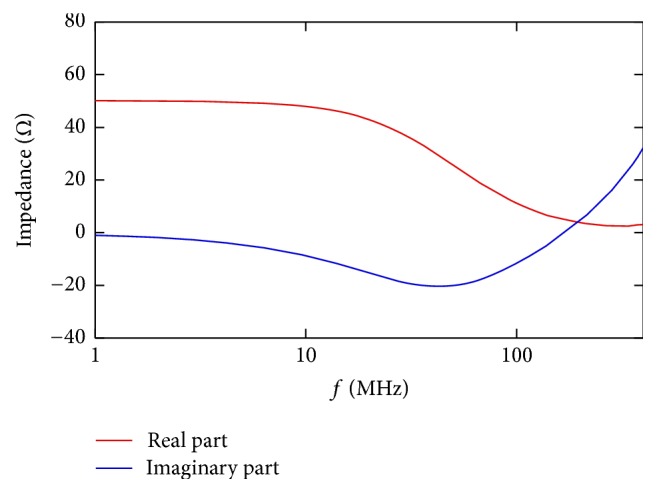
Impedance in terms of real and imaginary part of the holder with the cuvette placed and filled with a solution 0.25 S/m of conductivity.

As a first step in the analysis of the impedance behavior of the applicator, the real and the imaginary parts have been numerically evaluated. The results are reported in Figure 5 for the holder with the cuvette placed and filled with a solution 0.25 S/m of conductivity. As expected, at the lower frequency the resistive behavior is predominant, leaving for a capacitive one at the highest frequencies. It is worth noticing how this kind of coaxial connection reduces inductive parasitic effects, avoiding resonances within the range of the frequencies of interest (see resonance at around 350 MHz in [20]).

**Figure 6:**
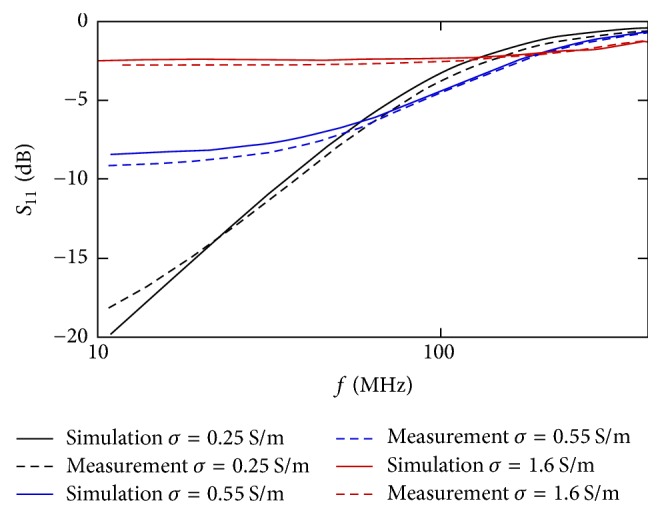
Measured (dashed lines) and simulated (solid lines) *S*_11_ parameters of the holder with the cuvette placed and filled with solution *σ* = 0.25 S/m (black line), 0.55 S/m (blue line), and 1.6 S/m (red line).

In the same frequency range the *S*_11_ has also been evaluated; the results are shown in Figure 6 (black solid line). From both the impedance and the *S*_11_ parameter, it is possible to see an acceptable frequency behavior up to 100 MHz (hence up to the first lobe of 10 ns pulsed electric field). The *S*_11_ value is ≤ −5 dB up to this frequency value.

At frequencies higher than 100 MHz the mismatch of the impedance cuvette is more evident and this limits the use of this kind of applicator only to pulses with duration ≥ 10 ns. The results achieved are comparable with the ones reported in [18, 20] for a cuvette with 4 mm gap distance.

### 3.2. Frequency Characterization for Different Solution Conductivities

Starting from the results obtained in [9], in which the importance of the external conductivity on the efficiency in liposomes nanoelectroporation has been reported, a characterization of the applicator behavior varying the conductivity of the solution placed inside the cuvette has been carried out. Simulations were run with different solution conductivities and compared with frequency domain measurements of the holder with the cuvette placed and filled with solutions at the same conductivities as the ones simulated (Figure 6).

The measurements and the simulations are in perfect agreement for all the considered solutions. As expected, the performances of the applicator strongly depend on the solution parameters, with best behavior for the less conductivity value (the best value according to the formula reported in [20]). Increasing the conductivity, the mismatch of the applicator increases, in particular for 0.55 S/m, with an *S*_11_ value always higher than −10 dB and for 1.6 S/m with values even higher than −5 dB. In this last case, the behavior of the structure is very close to a short circuit connection and hence with a high negative reflection coefficient when connected to a 50 Ω generator.

In order to better understand how this parameter affects the pulse transmission inside the cuvette, in the next paragraph the analysis of response in time domain of the cuvette holder with the cuvette placed and filled has been numerically performed.

### 3.3. Time Domain Characterization for Different Solution Conductivities

**Figure 7:**
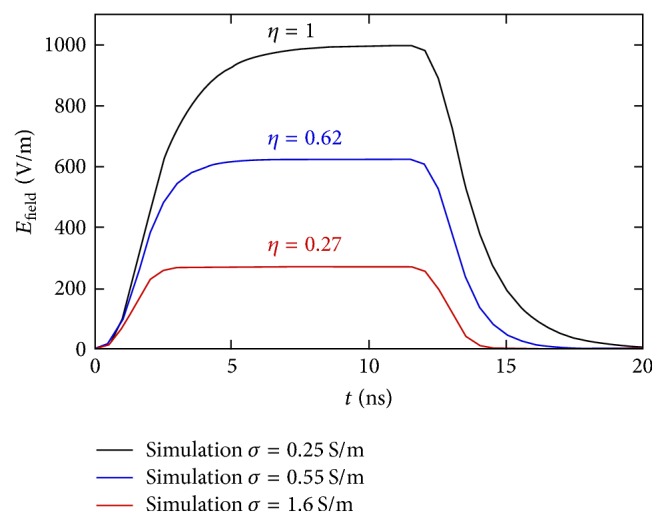
Electric field amplitude in the center of the cuvette gap. The efficiency value of the structure, *η*, defined as the ratio between the electric field obtained in the gap, in kV/m, and the applied voltage at the generator, in V, at different conductivity values are also reported.

The 10 ns pulse has been applied to the coaxial cable of the structure (Figure 3(c)). The cuvette has been filled with solutions at different conductivities and the transmitted signal has been evaluated in the center of the cuvette gap. In Figure 7, the results of transmitted electric field pulse for the different conductivity values are reported.

As expected from the frequency results, for the conductivity of 0.25 S/m, the amplitude of the transmitted pulse is 1000 V/m for 1 V input pulse. This is the maximum achievable value of electric field for 1 V applied to the 1 mm gap. The efficiency in transmission (*η*) is defined as the ratio between the electric field obtained in the gap, in kV/m, and the applied voltage at the generator, in V. In case of 0.25 S/m of conductivity, *η* is equal to 1. The rise and fall times of the transmitted pulse, as predictable by the *S*_11_ trend, appear distorted with a value around double (≈3 ns) with respect to the one imposed on the generator (1.5 ns). Increasing the conductivity value, the efficiency of the structure decreases assuming values of 0.62 and 0.27 for 0.55 S/m and 1.6 S/m, respectively.

Once the behavior of our applicator is completely characterized, a microdosimetry model of the liposomes is needed to derive all the necessary parameters to drive a final experiment of liposome solution exposed to a 10 ns pulse. In particular, microdosimetry should provide values of liposome poration for different experimental conditions, as vesicles dimensions or inner and outer conductivity, and with regard to the efficacy of the real structure that will be used for the experiments. Hence, the results of the model have to be combined with the information about the efficiency of the real applicator developed here.

### 3.4. Combining Experimental Results with Microdosimetry on Liposome

The microdosimetry model of the liposomes has been used to evaluate the electric field threshold value necessary to obtain the membrane liposome poration. In particular, 1 V has been considered as the threshold value for the electroporating transmembrane potential [8, 35] and the field threshold has been evaluated for different conductivities of the inner liposomes medium (*σ*_INT_) and of the external medium (*σ*_EXT_) ranging from 0.05 S/m to 1.6 S/m.

**Figure 8:**
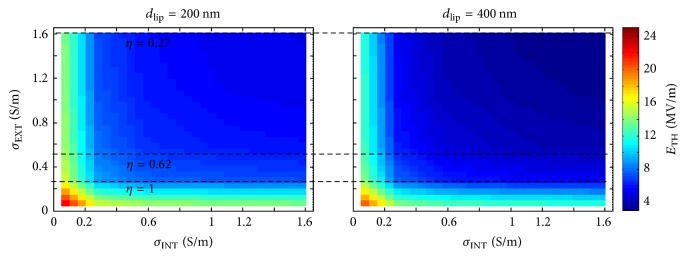
Electric field threshold for 200 nm and 400 nm liposomes poration changing the internal and external liposome conductivity.

In Figure 8, a 2D map of the electric field values necessary for liposomes electroporation with diameters of 200 nm or 400 nm is shown as a function of the external and internal conductivity. Considering the liposome suspended in external medium with a given conductivity value, it is possible to notice the influence of the inner conductivity along a line parallel to the *x* axis and crossing the *y* axis at the given external conductivity value. A liposome realized with a higher inner conductivity can be porated with a lower electric field value (e.g., for *σ*_EXT_ = 0.05 S/m and *σ*_INT_ = 0.05 S/m the threshold needed to reach the poration is about 22 MV/m, while for *σ*_EXT_ = 0.05 S/m and *σ*_INT_ = 1.6 S/m threshold is around 13.6 MV/m).

A dual situation occurs when the inner liposome conductivity is fixed and the external medium conductivity values change (i.e., a vertical line parallel to the *y* axis and crossing the *x* axis at a given inner conductivity value). Indeed a perfectly symmetric behavior is observed for the electroporation threshold when the inner or outer conductivities vary (maps in Figure 8 appear perfectly symmetric).

As expected, comparing the two different liposome dimensions (200 nm and 400 nm), the influence of the conductivities on the electric field threshold is the same, but the larger the dimension, the lower the field necessary for liposome membrane poration.

Considering from Figure 8 the effect on the threshold of external and internal conductivities and liposome dimension, the optimal conditions for liposome electroporation could simply be the highest inner and outer conductivity values and the larger liposome dimension.

However, as previously reported in this paper, the efficiency of our applicator in the delivering of pulse to the sample is inversely proportional to the outer conductivity (i.e., 1, 0.62, and 0.27 (kV/m)/V for 0.25, 0.55, and 1.6 S/m conductivity, respectively, black horizontal lines reported in Figure 8).

Hence, it is necessary to combine the data of Figures 7 and 8 in order to correctly predict the best practical experimental condition.

To weight the results of the microdosimetry model with the real experimental setup characteristics, we have taken into account not only the efficiency of the designed structure but also the capability of our generator in terms of the maximum output voltage it is able to generate. For the High Voltage Pulse Generator FPG 10-1NM10, we are able to provide a pulse with amplitude in the range of 2–10 kV, fixing our maximum capability to obtain 10 MV/m in 1 mm gap distance applying the highest intensity available.

**Figure 9:**
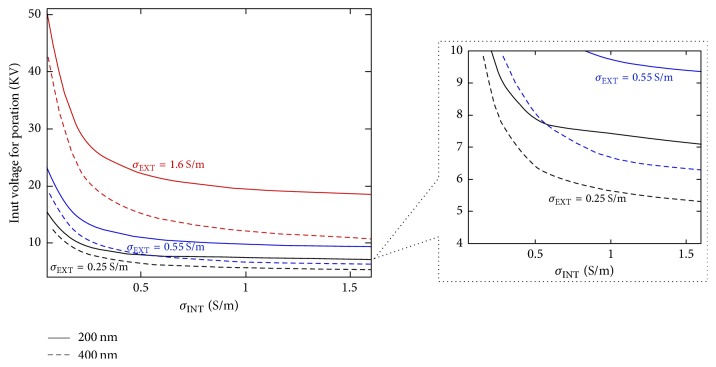
Input voltage needed at the generator weighted with the effective efficacy of the structure.

In Figure 9, the results in terms of voltage necessary at the generator in order to obtain the poration of liposomes are reported. The results are reported for liposomes with dimension of 200 nm (solid line) or 400 nm (dashed line) and for all the external conductivity values tested in the structure. The voltage results are weighted with the efficiency of the structure (*η* = 1, 0.62 and 0.27 ((kV/m)/V)) at the different conductivity values in order to understand the real amplitude needed during the experiments (Figure 9).

It can be noticed how the highest conductivity value (*σ*_EXT_ = 1.6 S/m), due to the very low efficiency of the structure, becomes disadvantageous for both the liposome dimensions. The 0.25 S/m condition with the higher efficiency produces the most convenient case in particular for liposomes with dimension of 400 nm. In the inset, a zoom up to 10 kV has been shown. It can be noticed that a 400 nm liposome, with an inner liposome conductivity of 0.55 S/m, immersed in a high conductivity medium is more easily electroporated than the 200 nm one in 0.25 S/m of conductive solution. However, looking at the inset, the most convenient experimental condition to demonstrate nanometer liposome poration is with a cuvette filled with a conductive solution of 0.25 S/m and an inner vesicle conductivity of 1.6 S/m for the 400 nm liposome. Under this configuration, a pulse of about 5 kV is enough to guarantee the electroporation phenomenon. With this kind of external conductivity also a 200 nm liposome could be electroporated, applying a higher voltage of 8 kV or 7 kV, respectively, for *σ*_INT_ of 0.55 S/m and 1.6 S/m.

## 4. Conclusions

The design and characterization of an exposure system with the capability to porate liposomes with nanometer dimensions using a 10 ns pulsed electric field have been carried out. This has been performed with an accurate system characterization taking into account both theoretical and technological issues. It represents the base of the integrated approach proposed in Figure 1, with a readjustment of the cycle proposed by Kitano [29, 30] for systems biology.

In the design of the structure the first step was to consider the features of the excitation and of the target. Since the excitation is a 10 ns pulsed electric field and the target is liposomes with 200 or 400 nm diameter, as suggested by the authors in [9], the use of a standard electroporation cuvette with 1 mm gap has been considered advantageous. The design of an appropriate cuvette holder structure proceeded, taking particular attention to the connection of the 1 mm gap electroporation cuvette to a 50 Ω High Voltage Pulse Generator. Further analysis demonstrated the capability of the proposed structure to deliver a 10 ns pulse without a significant signal distortion in particular for media with a 0.25 S/m of conductivity value. This structure is suitable for numerous experimental exposures, due to the indirect connection with the cuvette. Furthermore, the device guarantees a very strong mechanical stability and a good contact with the electrodes of the cuvette thanks to the Teflon box presence under it and as cover.

The structure has been completely characterized in frequency domain to understand its capability and performances in different experimental conditions. In particular, the analysis has been performed for different conductivity values of the solution inside the cuvette both with numerical models and measurements. A perfect agreement has been found between them. The higher the solution conductivity, the lower the efficacy of the structure and hence the lower the amplitude of the transmitted pulse to the cuvette.

In order to analyze the impact of the real performances of the device on the liposomes electroporation, a microdosimetry model of liposomes with 200 or 400 nm dimension has been reported. The simulations have been carried out considering different inner and outer liposome conductivities and the results have been combined with technological outcomes. Combining microdosimetry and technological information permits predicting that the optimal condition for a 400 nm liposome is with 0.25 S/m and 1.6 S/m for external and internal liposome conductivities, respectively, differently from the indications that one would follow on the basis of the microdosimetry alone (highest internal and external conductivity values). This discrepancy is due to the higher efficiency of the structure for the conductivity of 0.25 S/m. If during the experiments a change of some parameters becomes necessary, for example, different volumes of solution (keeping particular attention to avoid sparks in air) or a different value of inner or outer conductivities, a characterization with new simulations and measurements has to be performed in order to understand the real transmitted signal.

## Figures and Tables

**Table 1:** Electric and geometrical parameters.

Electrical and geometrical properties			
	*ε*_*r*_	*σ* (S/m)	Dimension (*μ*m)
External medium	67 [9, 26]	0.05–1.6	100–70
Liposome membrane	11.7 [9, 26]	1.1 × 10^−7^ [9, 26]	0.005 [9, 26]
Inner liposome	67 [9, 26]	0.05–1.6	0.2/0.4 [9]

Denzi et al. 2016 [9] and Merla et al. 2012 [26].
